# Spaceflight Influences both Mucosal and Peripheral Cytokine Production in PTN-Tg and Wild Type Mice

**DOI:** 10.1371/journal.pone.0068961

**Published:** 2013-07-10

**Authors:** Justin L. McCarville, Sandra T. Clarke, Padmaja Shastri, Yi Liu, Martin Kalmokoff, Stephen P. J. Brooks, Julia M. Green-Johnson

**Affiliations:** 1 Applied Bioscience Graduate Program and Faculty of Science, University of Ontario Institute of Technology, Oshawa, Ontario, Canada; 2 Università degil Studi di Genova, Dipartimento di Oncologia, Biologia e Genetica, Genova, Italy; 3 Istituo Nazionale per la Ricerca sul Cancro, Genova, Italy; 4 Atlantic Food and Horticulture Research Center, Agriculture and Agri-Food Canada, Kentville, Nova Scotia, Canada; 5 Bureau of Nutritional Sciences, Health Canada, Ottawa, Ontario, Canada; Ohio State University, United States of America

## Abstract

Spaceflight is associated with several health issues including diminished immune efficiency. Effects of long-term spaceflight on selected immune parameters of wild type (Wt) and transgenic mice over-expressing pleiotrophin under the human bone-specific osteocalcin promoter (PTN-Tg) were examined using the novel Mouse Drawer System (MDS) aboard the International Space Station (ISS) over a 91 day period. Effects of this long duration flight on PTN-Tg and Wt mice were determined in comparison to ground controls and vivarium-housed PTN-Tg and Wt mice. Levels of interleukin-2 (IL-2) and transforming growth factor-beta1 (TGF-β1) were measured in mucosal and systemic tissues of Wt and PTN-Tg mice. Colonic contents were also analyzed to assess potential effects on the gut microbiota, although no firm conclusions could be made due to constraints imposed by the MDS payload and the time of sampling. Spaceflight-associated differences were observed in colonic tissue and systemic lymph node levels of IL-2 and TGF-β1 relative to ground controls. Total colonic TGF-β1 levels were lower in Wt and PTN-Tg flight mice in comparison to ground controls. The Wt flight mouse had lower levels of IL-2 and TGF-β1 compared to the Wt ground control in both the inguinal and brachial lymph nodes, however this pattern was not consistently observed in PTN-Tg mice. Vivarium-housed Wt controls had higher levels of active TGF-β1 and IL-2 in inguinal lymph nodes relative to PTN-Tg mice. The results of this study suggest compartmentalized effects of spaceflight and on immune parameters in mice.

## Introduction

Multiple studies have demonstrated that spaceflight has both short and long term physiological effects, in human and animal subjects. These effects include bone loss, cardiovascular alteration (cardiac atrophy and change in heart rhythm), muscle loss, and immune dysfunction, among others [1]. Specifically, it has been reported that spaceflight modifies the immune system by altering cytokine production [2] and lymphocyte numbers, specifically T cells [3]. Although the effects of spaceflight on the immune system have been the focus of several studies, there is currently a lack of knowledge with respect to how the gut microbiota is altered in spaceflight and under microgravity conditions, and the consequent impact on the immune system. Therefore, colonic contents were analyzed to determine whether effects on the gut microbiota could also be detected. The gut microbiota is known to affect both mucosal and systemic immunity, including IgA-secreting plasma cells, CD4^+^ T cell populations and antimicrobial peptide secretion, as well as altering intestinal epithelial cell cytokine expression [4], [5], [6], [7]. Therefore, changes in the gut microbiota during spaceflight and under microgravity conditions may also affect the immune system. Transgenic mice over-expressing pleiotrophin under the human bone specific osteocalcin promoter (PTN-Tg) and wild type (Wt) mice were also used for this study [8], allowing for additional insight into effects of this transgene on the immune system under both flown and ground control conditions. PTN is part of the midkine family of heparin-binding growth factors, and is involved in osteogenesis and bone formation, as well as neurogenesis, cell proliferation and inflammation [9], [10]. Decreased bone density and bone mass are issues associated with space flight [8]. The PTN-Tg mouse model was selected for the leading study in the MDS mission flown aboard the ISS, in order to determine whether PTN over-expression would provide protection from bone loss due to microgravity [8], [11]. The aim of present study was to examine effects of long-term space flight on immune parameters and the gut microbiota, through participation in a tissue-sharing program [11]. However, as little is currently known about the effects of PTN overexpression on immune parameters, the use of both wild type and PTN-Tg mice in the MDS system also provided a novel opportunity to examine effects of PTN overexpression and of long-duration space flight on the immune system.

While numerous effects of spaceflight on the immune system have been reported, the potential contribution of spaceflight-associated effects on the gut microbiota to such changes has received little attention to date. Increasing evidence points to numerous interactions of the gut microbiota with the immune system [12], and determining effects of spaceflight on the gut microbiota, and the subsequent outcomes for immune activity will be valuable for optimizing long-term space flight conditions. To gain insight into these interactions utilizing a limited quantity and range of tissues, our focus was on cytokines previously reported to be affected by spaceflight, important for immune regulatory or effector activity, and responsive to gut microbiota changes: TGF-β1 and IL-2 [13], [14].

TGF-β1 is a multifunctional cytokine that plays an integral role in adaptive immunity, mucosal immunoregulation as well as bone formation, osteoblast proliferation and differentiation, and is influenced by the gut microbiota [13], . TGF-β1 is often considered a regulatory cytokine, involved in intestinal immune homeostasis and control of T cell activity and differentiation [19]. Previous studies have demonstrated that TGF-β1 is down-regulated in certain tissues under microgravity conditions, including reduced TGF-β1 mRNA levels in bone and in osteoblasts, as well as decreased osteoblast TGF-β1 production [17],[20],[21],[22]. While little is currently known about interactions between PTN-Tg and TGF-β1, concurrent upregulation of PTN and TGF-β1 has been reported in chlorhexidine gluconate (CG)-induced fibrosis [9]. Given the role of TGF-β1 in osteoimmunology and the previously documented effects of microgravity on TGF-β1 expression, we also hypothesized that mice overexpressing pleiotrophin may have differential TGF-β1 production under both space flight and ground conditions. IL-2 is essential for T cell proliferation and differentiation of specific T-cell subsets, including the differentiation of naïve CD4^+^ T cells into T helper 1 cells and promotion of CD8^+^ T cell proliferation. However, it plays other roles, such as increasing antibody secretion from plasma cells [23]. Previous studies have indicated IL-2 production decreased in astronauts following spaceflight. Specifically, lower levels of post-flight IL-2 production by CD4^+^ and CD8^+^ T cells have been observed [2]. The same pattern has been shown in rats following spaceflight, where the percentage of splenic T cells (TCR^+^/CD4^+^) was reduced in comparison to ground controls [24]. Quantification of the cytokines TGF-β1 and IL-2 in mice that have had prolonged exposure to spaceflight would thus provide insight into the consequences for T cell activity and differentiation in both PTN-Tg and Wt mice, as well potential effects on bone formation, which are two key issues for astronaut health during long-duration spaceflight.

In this component of the MDS Experiment on the ISS, we examined effects on selected mucosal and systemic immune parameters and also attempted to assess the effects on the gut microbiota by examining the colonic community in flight mice, in the corresponding ground and vivarium controls, and in transgenic versus Wt mice. The lack of gut community diversity made inferences into interactions of gut microbiota and the immune system difficult, as did the limited number of mice surviving the flight. However, due to the unique nature of the MDS experiment and inability to readily conduct additional replicates, we carried out analysis of the TGF-β1 and IL-2 levels in the available tissue and analysis of the colonic microbiota community, and provide here an observational analysis of the findings. These results may provide further insight not only into effects of spaceflight on the immune system in systemic and mucosal compartments, but also into design issues for future studies aiming to examine effects of long term space flight on the gut microbiota, and associated effects on host parameters.

## Results

Full details regarding the MDS Experiment and flight opportunity have been previously described in Cancedda *et al.,* 2012 [11]. Unfortunately, there was a limited sample number for this experimental analysis due to the poor survival rate of the space flight mice. Upon returning to earth, there were 2 surviving PTN-Tg mice and a single Wt mouse. The ground control sample numbers were adjusted to match those of the flight mice. Tavella *et al*., 2012 have previously outlined details concerning consequences and timing of rodent death for both flight mice and ground controls [8]. The limited sample number was not ideal for comparing the effects of long-term space flight on cytokine levels between PTN-Tg and Wt groups, and statistical analysis was only possible for the comparing these two groups in the vivarium controls. The observations made throughout this portion of the MDS study do however contribute further to the overall investigation of effects of long term space flight made possible through a tissue-sharing program. The findings from this unique experimental situation may provide groundwork for future studies aboard the ISS, and illustrate certain design issues that will be important for studies examining effects of spaceflight on the gut microbiota and interactions with host parameters. All animals involved in the trial gained weight by experiment completion, as previously reported [11]. Gross necropsy of the colonic and lymphoid tissues did not reveal any abnormal changes in the gastrointestinal structure or mucosal tissue.

### Effects of Long Term Space Flight on Colonic TGF-β1 and IL-2 in Wt and PTN-Tg Mice

Both TGF-β1 (active and total) and IL-2 were quantified within the colonic tissue of PTN-Tg and Wt mice, in order to observe the effects of long-term space flight on cytokine production at the mucosal level (Table 1). PTN-Tg mice showed variable levels of colonic IL-2 in the ground control, vivarium and flight conditions compared to IL-2 levels in Wt colonic tissue, which were undetectable in all but one mouse. The highest levels of active and total TGF-β1 were also observed in the PTN-Tg ground controls, and one of the Vivarium-housed mice. PTN-Tg and Wt ground control mice were compared to their flight counterparts in order to elucidate the effect of spaceflight on this parameter of mucosal immunity. TGF-β1 (active and total) levels in PTN-Tg mice were lower under flown conditions, and were undetectable in the Wt flight mouse, in contrast to the much higher level of total TGF-β1 in the Wt ground control. Both colonic IL-2 and active TGF-β1 were below the level of quantification for the ground control Wt mouse and the flown WT mouse. Statistical analysis was only possible for comparison of the colonic TGF-β1 levels in Wt vivarium control and PTN-Tg vivarium control mice, and no significant difference was observed.

**Table 1:** Cytokine levels within the colonic tissue of PTN-Tg and Wt mice under flight, ground control and vivarium conditions.

Cytokine	Wt GroundControl	PTN-Tg Ground Control	Wt Flight	PTN-Tg Flight	Wt VivariumControl	PTN-Tg Vivarium Control
**IL-2 (pg/g)**						
1^1^	–3	BLQ^2^	–	1.08	BLQ	173.01
2	BLQ	102.80	BLQ	7.08	BLQ	22.13
3	–	–	–	–	79.67	9.47
mean ± SEM				4.08±3.00		68.20±52.53
**Active TGF-β1 (pg/g)**						
1	–	19.98	–	1.28	BLQ	192.27
2	BLQ	92.23	BLQ	9.38	BLQ	2.57
3	–	–	–	–	58.09	2.77
mean ± SEM		56.11±36.13		5.33±4.05		65.87±63.20
**Total TGF-β1 (pg/g)**						
1	–	2715.79	–	64.34	15.21	2168.24
2	315.20	2648.98	BLQ	181.58	217.93	623.77
3	–	–	–	–	94.83	240.05
mean ± SEM		2682.39±33.41		122.96±58.62	109.32±58.97	1010.69±589.28

1 Cytokine levels of individual mice are shown per gram of colonic tissue.

2 Below level of quantification (BLQ).

3 Deceased mice.

### Effects of Long Term Space Flight on Inguinal Lymph Node Cytokine Levels of Wt Mice Relative to PTN-Tg Mice

Wt mice appeared to be affected by flight more so than their transgenic counterparts with respect to inguinal node cytokine production, but additional testing would be required to confirm this observation. As shown in Table 2, the lowest inguinal IL-2 levels were observed in the Wt flight mouse compared to the PTN-Tg flight mice. The Wt and transgenic ground control mice had similar IL-2 levels. Flight appears not to have had any discernable effects on inguinal node IL-2 levels in the transgenic mice. The flown PTN-Tg mice compared to the PTN-Tg ground control mice had similar inguinal IL-2 levels.

**Table 2:** Cytokine levels in the inguinal lymph nodes of PTN-Tg and Wt mice under flight, ground control and vivarium conditions.

Cytokine	Wt Ground Control	PTN-Tg Ground Control	Wt Flight	PTN-Tg Flight
**IL-2 (ng/g)**				
1^1^	–2	8.54	–	3.88
2	7.17	5.08	2.41	9.13
3	–		–	–
mean ± SEM		6.81±1.73		6.51±2.63
**Active TGF-β1 (ng/g)**				
1	–	21.27	–	10.41
2	21.22	6.41	11.73	22.87
3	–	–	–	–
mean ± SEM		13.84±7.43		16.64±6.23
**Total TGF-β1 (ng/g)**				
1	–	72.22	–	20.25
2	100.90	33.56	19.96	61.57
3	–	–	–	–
mean ± SEM		52.89±19.33		40.91±20.66

1 Cytokine levels of individual mice are shown.

2 Deceased mice.

**Figure 1:**
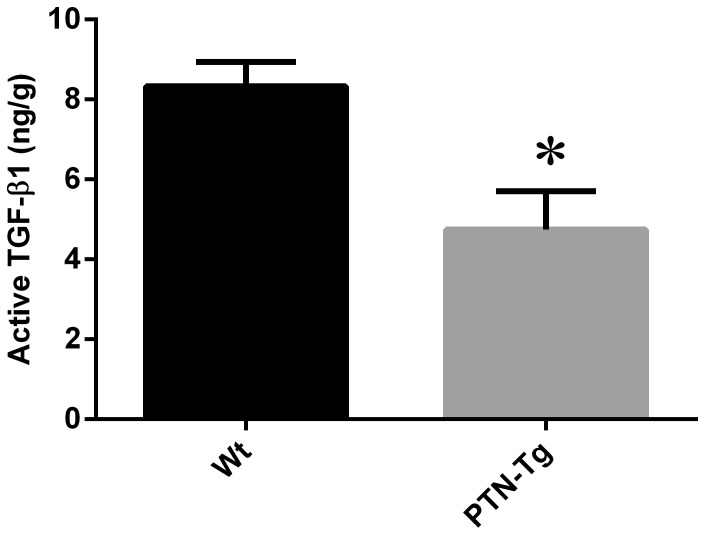
Active TGF-β1 (ng/g) levels in inguinal lymph node from Wt and PTN-Tg vivarium housed mice. TGF-β1 was quantified from homogenized inguinal tissue of Wt and PTN-Tg vivarium housed mice by ELISA. The PTN-Tg mice had lower amounts of active TGF-β1 compared to the Wt. * Indicates statistical significance (p = 0.05) by unpaired Welch corrected t test.

**Figure 2:**
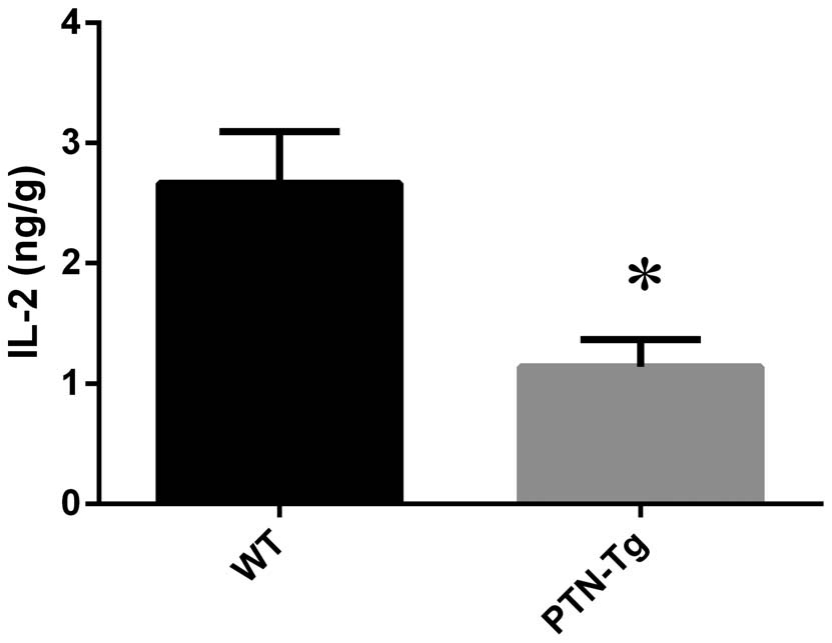
Comparison of IL-2 (ng/g) in inguinal lymph nodes from Wt and PTN-Tg vivarium- housed mice. IL-2 was quantified from homogenized inguinal tissue of Wt and PTN-Tg vivarium housed mice by ELISA. The PTN-Tg mice had lower amounts of IL-2 compared to the Wt. * Indicates statistical significance (p = 0.05) by unpaired Welch corrected t test.

Levels of TGF-β1 in the inguinal lymph nodes of Wt mice compared to PTN-Tg mice were different under flight conditions. The percentage of active TGF-β1 was higher in the Wt flight mouse compared to the PTN-Tg flight mice (Table 3). The Wt and PTN-Tg ground control mice had similar percentages of active TGF-β1. Vivarium-housed PTN-Tg mice had significantly lower levels of active TGF-β1 (Figure 1) and IL-2 (Figure 2) compared to Wt mice. Though statistical comparisons were not possible against ground or flown mice, the pattern of active TGF-β1 and IL-2 in the inguinal nodes of the vivarium-housed mice appears reversed compared to flown mice, but similar to ground control mice.

**Table 3:** Percent active TGF-β1 in inguinal and brachial lymph nodes of PTN-Tg and Wt mice under flight, ground control and vivarium conditions.

Tissue^1^	Wt Ground Control	PTN-Tg Ground Control	Wt Flight	PTN-Tg Flight	Wt VivariumControl	PTN-Tg Vivarium Control
% Active TGF-β1 (inguinal lymph node)	21.03^a^	24.28b^b^±5.18	58.77^a^	44.26^b^±7.13	45.72^c^±10.20	48.50^c^±11.87
% Active TGF-β1 (brachial lymph node)	57.46^a^	41.06^b^±11.94	46.31^a^	63.92^b^±14.56	68.94^c^±5.44	37.97^c^±10.74

1 Percent active TGF-β1 levels in colonic tissue were negligible.

a n = 1,

b n = 2,

c n = 3.

### Production of TGF-β1 and IL-2 in Brachial Lymph Nodes of Wt and PTN-Tg under Spaceflight and Ground Control Conditions

The brachial lymph node homogenate was investigated for effects of the PTN-Tg mutation and of spaceflight on peripheral cytokine production by quantifying the levels of IL-2 in addition to total and active TGF-β1 (Table 4). IL-2 concentrations were lower in the brachial lymph nodes of both PTN-Tg and Wt mice under flight conditions when compared to the ground controls. The level of IL-2 in the brachial nodes of the Wt flight mouse was higher than that observed in either of the PTN-Tg flight mice, similar to the pattern seen in the brachial nodes of the ground control mice. However, there was no statistically significant difference observed in the brachial lymph node IL-2 concentrations of the Wt and PTN-Tg mice housed in vivarium conditions.

**Table 4:** Cytokine levels in the brachial lymph nodes of PTN-Tg and Wt mice under flight, ground control and vivarium conditions.

Cytokine	Wt Ground Control	PTN-Tg Ground Control	Wt Flight	PTN-Tg Flight	Wt VivariumControl	PTN-Tg Vivarium Control
**IL-2 (ng/g)**						
1^1^	–2	12.11	–	3.87	4.61	7.37
2	19.03	10.34	10.99	7.13	6.54	4.87
3	–	–	–	–	9.99	6.51
mean ± SEM		11.23±0.89		5.5±1.63	7.05±1.57	6.25±0.73
**Active TGF-β1 (ng/g)**						
1	–	18.05	–	32.08	6.15	16.22
2	47.33	20.42	28.92	38.50	28.63	4.00
3	–	–	–	–	40.37	9.55
mean ± SEM		19.24±1.19		35.29±3.21	25.05±10.04	9.92±3.53
**Total TGF-β1 (ng/g**)						
1	–	61.99	–	41.41	9.28	29.23
2	82.36	38.53	62.45	79.63	46.79	21.69
3	–	–	–	–	50.82	23.90
mean ± SEM		50.26±11.73		60.52±19.11	35.63±13.23	24.94±2.24

1 Cytokine levels of individual mice are shown.

2 Deceased mice.

Total TGF-β1 levels were lower in brachial nodes of PTN-Tg mice than the Wt mouse in ground control conditions, but the lowest total brachial TGF-β1 levels were observed in vivarium-housed mice. The flown Wt mouse exhibited decreased levels of active and total TGF-β1 in flight conditions when compared to levels in the Wt ground control. Conversely, the brachial nodes of PTN-Tg mice had higher levels of both active and total TGF-β1 in the flight condition when compared to levels in ground controls. Notably, the brachial lymph node concentrations of active TGF-β1 of the PTN-Tg mice were higher in flown conditions than in the Wt counterpart, as was the percent active TGF-β1. Under flight conditions, the percent active TGF-β1 was lower in the Wt mouse and higher in the PTN-Tg mice compared to levels in ground controls, although statistical analysis was not possible due to the limited sample size.

Statistical analysis was only possible for mice housed under vivarium conditions; however the difference in TGF-β1 (active, total and percent active) in the brachial lymph nodes between PTN-Tg and Wt mice was not statistically significant.

### Gut Microbiota and Spaceflight

**Figure 3:**
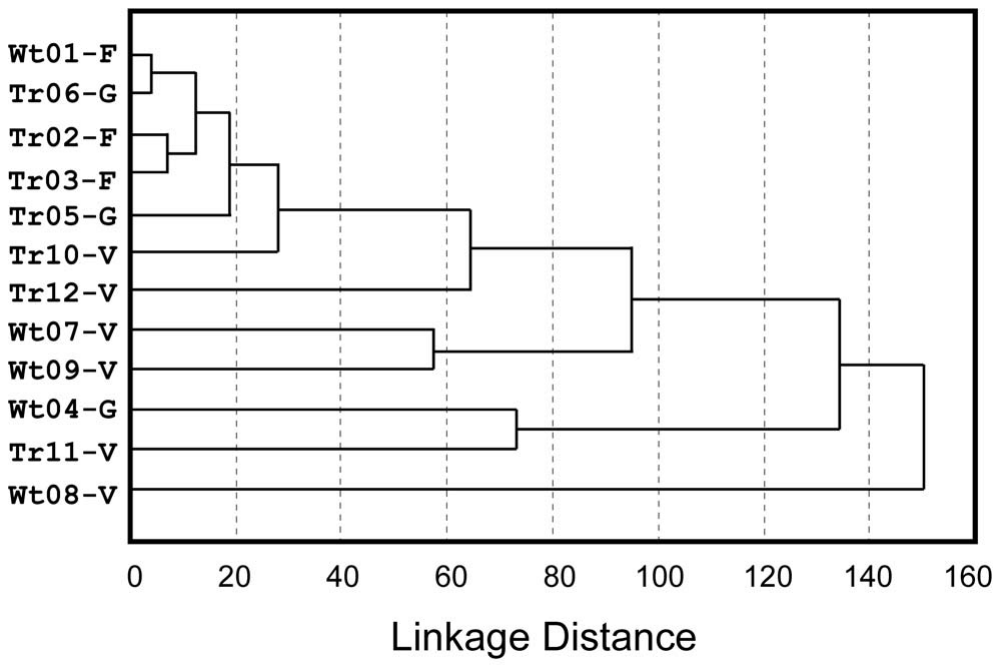
Dendrogram comparing colonic communities among mice (Wt: wild type or Tg: transgenic) subjected to space exposure (F) the corresponding MDS ground controls (G) or mice from the vivarium (V).

Colonic contents collected from mice subjected to spaceflight, the corresponding MDS ground control, and from vivarium-housed mice were examined in order to determine whether spaceflight affected the gut community. All of these samples were liquid and contained no visible particulate material. Analysis of pyrosequencing data yielded a total of 137 phylotypes shared among these samples. Cluster analysis comparing the colonic community among each sample indicated little difference in the microbiota of mice exposed to spaceflight versus those in the corresponding ground control MDS system (Figure 3). In all cases, these communities were dominated by a single phylotype homologous with *Lactobacillus reuteri*, which encompassed 84–99% of the total phylotypes within each of these samples. Colonic samples from the vivarium-housed mice were richer and mostly clustered further away, but were also dominated by this phylotype, although to a lesser degree (10–60% of total phylotypes). A common feature was the uneven diversity within each sample.

## Discussion

The present study investigated the effects of long-duration spaceflight on selected immune system parameters in both Wt and PTN-Tg mice. While considerable evidence points to changes in the efficacy of the immune system during spaceflight [2], [3], it is currently not known whether these changes are to any extent due to flight-induced alterations in the gut microbiota. The MDS experiment provided a unique opportunity not only to determine effects of long-duration spaceflight on the immune system, and potentially on the gut microbiota, but also to examine these effects in the context of the PTN-Tg mutation.

TGF-β1 is a highly conserved, pleotropic cytokine with varied effects on numerous cell types, including regulation of B cell differentiation and class switching, attenuation and regulation of Natural Killer (NK) cell activity and regulation of dendritic cells (DC) maturation and antigen-presenting activity [15], [25], [26]. Immunoregulatory activity of TGF-β1 also reflects its profound impact on T cell activity and differentiation [27], and TGF-β1 is integral in maintaining intestinal immune homeostasis [15], [19]. For example, TGF-β1 is involved in conditioning mucosal DCs to a phenotype supporting differentiation of T Helper (TH) cells to the regulatory T cell (T_reg_) phenotype, which are in turn essential for maintaining intestinal immune homeostasis and controlling excessive inflammatory responses at the mucosal interface [28], [29]. TGF-β1 is also a class switching factor for B cells, promoting production of IgA, the predominant mucosal antibody isotype [30].

In addition to its integral role in the immune system, TGF-β1 also plays a role in wound healing and fibrosis, and bone formation [16], [31]. Bone loss is a well-documented issue associated with spaceflight, and decreased bone formation has been identified as a contributing factor [32], [33]. TGF-β1 is involved in induction of bone formation, and TGF-β1 mediated signalling is involved in skeletal development [34]. Compartment-specific reduction in skeletal TGF-β1 mRNA levels following spaceflight have been observed in rats [20], suggesting decreased osteoblast activity. Decreased post flight levels of TGF-β1 and TGF-β2 mRNA in cultured human fetal osteoblastic cells [21] and reduced TGF-β1 production by rat osteoblasts following 4 and 5 days of flight, relative to ground controls have also been reported [22]. Microgravity-induced decreases in osteoblast TGF-β1 mRNA levels have been shown to return to normal under artificial onboard gravity conditions [17], further illustrating the effects of space flight on this cytokine.

In this component of the MDS study, the effect of spaceflight on mucosal immunity was examined by quantifying IL-2 and TGF-β1 levels within colonic tissue of flight and ground control mice, as well as in vivarium controls. Our results suggest a spaceflight associated decrease in total TGF-β1 levels in colonic tissue of PTN-Tg mice and the Wt mouse. Given the role of TGF-β1 in T_reg_ induction and development [28], the decrease in total TGF-β1 could be associated with declining T cell numbers, as observed in other spaceflight studies [35]. These observed changes in mucosal TGF-β1 production could be the consequence of external factors such as microgravity, although the potential effect of gut microbiota interactions with the mucosal immune system cannot be ruled out.

Activation of T cells requires extensive cell-cell communication and signal transduction, effected through appropriate ligand-receptor recognition. An important cytokine which activates resting T cells is IL-2, secreted by activated TH cells. In this study, it was observed that the Wt mouse under flight conditions produced lower IL-2 levels in systemic tissues (brachial and inguinal lymph nodes) compared to its ground control counterpart. Similar results with respect to IL-2 production were observed by Gridley *et al.* (2009) when splenocytes from flight mice were activated by anti-CD3 stimulation [3]. They postulated that the lowered IL-2 production could be due to alterations in T cell signal transduction [3]. A previous study attempted to reverse the negative effects of spaceflight on T cell populations in rats by administering pegylated interleukin-2 (PEG-IL-2), however, this treatment was unable to oppose the effects of spaceflight [36]. This suggests that the effects of spaceflight on T cell populations are not solely due to decreased IL-2 production. Our study did not investigate T cell populations specifically, however, due to the integral role of IL-2 in T cell activation, diminished levels of IL-2 could reflect reduced T cell activation and proliferation in space-flight conditions.

The percentage of active TGF-β1 in the inguinal nodes was somewhat higher in the Wt flown mouse compared to the ground control, but an opposite pattern emerged in the brachial lymph nodes. Microgravity shifts the normal head-to-foot hydrostatic gradient, leading to fluid retention in the upper extremities and limiting pressure to the cervical areas [37]. Changes in lymph recirculation under microgravity conditions could influence cellular migration, potentially resulting in alterations in cell populations within peripheral lymph nodes. If fluid shift effects of microgravity influence T cell populations in lymph nodes, they may also account for the decreased levels of IL-2 observed in both inguinal and brachial lymph nodes of the flight mice, as similar results have been observed for mice under simulated microgravity conditions [38].

Exposure to low gravity plays a role in the inhibition of lymphocyte proliferation due to its impact on cytokine secretion, T cell locomotion and differential structuring of lymphocyte cytoskeletal elements [39]. Thus prolonged spaceflight may lead to suppressed immune responses. Though difficult to ascertain with certainty due to the small experimental sampling pool, it appears that immune suppression may have occurred in Wt mice during spaceflight based on the pattern of TGF-β1 and IL-2 production in systemic locations. This may be due to a multitude of reasons, including lower T cell proliferation due to selective changes in adhesion molecules expressed on lymphocytes during spaceflight [40]. Other possibilities include thymic atrophy [41] leading to lower T cell populations or altered T cell populations due to changes induced by flight stress, radiation and low gravity [3], and differential cell trafficking between tissues due to microgravity induced fluid shift [42].

The PTN-Tg mice in this study were over-expressing pleiotrophin under control of the human bone-specific osteocalcin promoter [43]. The impact of this transgene in spaceflight has never been investigated prior to this long term (91 day) flight using the MDS system on the ISS, where PTN-Tg expression exerted a protective effect against microgravity-induced bone loss through increased osteoblast activity [8], [11]. Within the colon, a mucosal tissue, there was a decrease in TGF-β1 (active and total) and IL-2 in PTN-Tg flight mice relative to ground control mice. This same pattern was also observed with total TGF-β1 levels in the Wt flight mouse. Therefore, although the PTN-Tg mice expressed higher levels of TGF-β1 both on the ground and in flight, the transgene had little effect on preventing cytokine level decreases in flight. Interestingly, PTN-Tg flight mice showed no decrease in inguinal node IL-2 or TGF-β1 levels or in brachial node TGF-β1 levels, relative to PTN-Tg ground control mice, in contrast to the lower levels observed in these systemic tissues of the Wt flight mouse. This could potentially be due to the transgene increasing bone mass, and perhaps the efficiency of producing lymphocytes from bone marrow. Previously, it has been observed that microgravity negatively affects expansion and differentiation of stem cells into lymphocyte and monocytes [44]. If this were the case, the effects of spaceflight on T cell populations in PTN-Tg mice could be lower than those in Wt mice.

The effect of over-expressing PTN under the osteocalcin promoter on immune parameters has received little attention to date. In this study, it was observed that the levels of IL-2 in both lymph node tissues of Wt and PTN-Tg were lower in vivarium conditions when compared to ground control counterparts. These differences in vivarium in relation to ground control conditions may be due to the nature of the housing environment, or to decreased stressors. Reduced production of TGF-β1 in PTN knockout mice in response to CG treatment has been previously reported [9]. However, higher levels of active TGF-β1 were quantified in the inguinal lymph node tissues of vivarium-housed Wt mice, suggesting that PTN overexpression may decrease TGF-β1 production or activation, suggesting a complex relationship between these mediator molecules. It has been previously demonstrated that adult mice with PTN over-expression have long term impairment of bone healing and bone strength [45]. Since TGF-β1 has strong influences in bone remodelling and structure [46], decreases in levels of this cytokine may lead to decreased bone strength. It would be interesting to determine whether the lower levels of active TGF-β1 observed in the vivarium PTN-Tg mice are directly caused by PTN over-expression.

The observation that the collected colonic contents contained no visible particulate material and that the communities consisted primarily of a single *L. reuteri* strain made it impossible to assess the potential impacts of spaceflight on the gut community in any of the mice. Gut transit time in mice is very fast, having been estimated at approximately 4.5 hours [47]. One possible explanation is that the mice were in a “fasted” state at the arrival to the Space Life Science Laboratory at the Kennedy Space Center (KSC). This is deduced from the fact that the MDS payload was switched to the “survival” mode about three days before the landing at KSC [11], and mice tend to finish eating their ration shortly after 5 g of food is dispensed by the food delivery system [48]. Even if the mice had consumed food between the arrival to SLSL and the time of dissection, the food digest could have been retained in the stomach or small intestine, resulting in the observed scant colonic content. Unfortunately neither stomach nor small intestine, nor their contents were made available to us. Our results illustrate the potential for design factors such as timing of feeding, to influence analysis of gut microbiota diversity. Design of future studies to analyze the impact of spaceflight on the gut microbiota will need to integrate strategies to deal with the challenges and constraints imposed by such elements as housing design, feed delivery and animal management during landing.

In summary, while spaceflight-associated differences were observed in colonic tissue and systemic lymph node levels of IL-2 and TGF-β1, direct correlations with effects on the gut microbiota could not be made, due to the scant colonic content. These immune alterations could result from a variety of factors including impeded movement of lymphatic fluids, effects of microgravity on T cell populations, or from spaceflight-associated effects on the gut microbiota.

Future studies could potentially address these elements, as well as investigate the site of cytokine production. It was not possible to perform histopathology or immunohistochemistry experiments in this study since the limited sample number and diminutive proportions only provided enough tissue for homogenate cytokine analysis. Such experiments could provide insight as to whether immunological adaptations to space flight took place in the lymphoid tissues. However, it must be noted that changes in immune parameters, and potentially even in the gut microbiota may not be due to microgravity itself, but instead due to other elements such as changes in diet, exposure to radiation or even psychological stressors and the resulting neuro-immune interactions [2]. Further study would be valuable in order to determine the extent of the effects on cytokine production and the roles played by these factors relative to direct effects of microgravity in both Wt and PTN-Tg mice.

## Materials and Methods

### Animals and Animal Habitat

Details regarding the mouse drawer system and exposure of C57BL/10J Wt and PTN-Tg mice to zero gravity on the ISS have been previously described in “The Mice Drawer System Experiment and the Space Endurance Record-Breaking Mice” (2012) *PLoS Collections* [8], [11], [49], [50], [51]. Mice were 8 weeks old at launch. Three Wt and three PTN-Tg mice were housed in the MDS system, and delivered via the Space Shuttle Discovery (Space Transport System (STS)-128 mission) to the ISS on August 28^th^, 2009. The surviving 1 Wt and 2 PTN-Tg mice were returned to earth after 91 days, via the Space Shuttle Atlantis (STS-129 mission). The authors participated in a tissue sharing process, receiving specific samples (brachial and inguinal lymph nodes, colon and colonic contents) at the end of the flight period. During the tissue collection, a Biospecimen Reporting for Improved Study Quality (BRISQ) checklist was employed to ensure specimen uniformity. Animal care protocols were approved by the University of Ontario Institute of Technology (UOIT) Animal Care Committee, the American Institutional Animal Care and Use Committee (IACUC), Ethics Committee of the Animal Facility of the National Institute for Cancer Research (Genova, Italy) and by the Public Veterinary Health Department of the Italian Ministry of Health.

### Sample Preparation and Storage

The inguinal and brachial lymph nodes and colonic tissue were removed by dissection; flash frozen, shipped immediately to UOIT and stored at -80°C. To prepare the samples, tissues were weighed and suspended in phosphate buffered saline with 0.5% protease inhibitor cocktail and DMSO (P8340– Sigma, St. Louis, MO). Tissues were homogenized using a Tissuemiser homogenizer (Fisher Scientific, Hampton, NH) and centrifuged (16 100×*g*, 30 min). The supernatants were collected and the centrifugation process was repeated until tissue and fat were no longer prevalent in the supernatant. The final products were stored at -80°C for future assay.

Contents contained within the colon of each mouse (∼100 mg/mouse) were recovered during dissection and frozen at −80°C. DNA was recovered from each sample by first grinding in liquid nitrogen then purified using the QIAamp DNA stool mini kit (Qiagen Inc, Mississauga, ON). Bacterial tag-encoded FLX amplicon pyrosequencing was performed as previously described [52] using the forward primer F44 [53] and the reverse primer R519 [54]. Initial sequence processing was carried out using Esprit [55]. Phylotypes within each sample were screened to identify suspected chimeric sequences determined using Chimera-Slayer [56] as implemented through Mothur [57]. Suspected chimeric sequences and phylotypes encompassing <2 reads per sample were removed. Phylotypes within each sample were trimmed to an equal length, aligned and clustered at a 0.03% sequence divergence using Mothur. Neighbour-joining trees were generated using seaview [58] with Jukes Cantor correction [59] and 1000 iterations. Additional sequences aligning within a <3% sequence divergence were further binned together. This was repeated until the core phylotypes within each sample set were identified. Shared phylotypes were identified by pooling the core phylotypes across all samples and processed as carried out for each sample. Phylotype classification was determined using the Seq-match program available through the ribosome data project [60].

### TGF-β1 and IL-2 Cytokine Quantification

Both TGF-β1 and IL-2 were quantified by enzyme linked immunosorbent assay (ELISA), using kits DY240 and DY402 respectively, (R&D Systems, Minneapolis, MN) and high affinity protein binding ELISA plates (Greiner Bio-One). Microcentrifuge tubes were siliconized with Sigmacote® (SL2 - Sigma, St. Louis, MO) prior to preparation of dilutions and activation of samples for TGF-β1 quantification. Levels of both active and total TGF-β1 were determined. Activation of TGF-β1 samples to remove the Latency Associated Protein (LAP) was carried out using standard acidification protocols as outlined by R&D Systems. The optical density was determined using a Synergy HT plate reader (BioTEK, Winooski, VT) at 405 nm.

### Statistical Analysis

Statistical analyses were performed in InStat 3 (GraphPad Software, La Jolla, CA) using unpaired two-tailed T-tests. However, statistical analysis was not possible for all ground controls and space flight mice due to the small sample size which survived flight. For cluster analysis, phylotype frequency distributions were first calculated for each colonic sample with abundance normalized by expressing the contribution of each individual phylotype as a percent of the total sample. This data was pooled then subjected to cluster analysis using Statistica (Statsoft, Tulsa, OK) using city block distances [61].
